# Intraocular twin cysticercosis

**DOI:** 10.3205/oc000226

**Published:** 2023-09-29

**Authors:** Rohini Grover, Abhishek Varshney, Supreet Juneja, Upma Awashti, Sonali R. Singh

**Affiliations:** 1 Department of Vitreoretina, CL Gupta Eye Institute, Moradabad, India; 2 Department of Vitreoretina, Sunetra Eye Care Centre, Ghaziabad, India

**Keywords:** twin cysticercosis, retinal detachment, B-scan, pars plana vitrectomy

## Abstract

A 20-year-old vegetarian male presented with a history of painful diminution of vision in the right eye for the past month. The patient had dense vitritis. B-scan ultrasonography (USG) revealed two cysts with scolices, one subretinally along with retinal detachment and another one in vitreous cavity. Orbital USG revealed no cystic lesions in the orbit or extraocular muscle. He underwent 23 gauge pars plana vitrectomy. Both intravitreal and subretinal cysts were cut and aspirated using cutter and removed from the eye, and silicon oil was injected. Postoperatively he was started on oral steroids and advised to maintain prone positioning for two weeks. At two months his best corrected visual acuity (BCVA) in the right eye was 20/125 with silicon oil in situ.

## Background

Cysticercosis in humans is caused by tissue infestation of the cysticercus larva of the pork tape worm, *Taenia solium* [1]. Cystercerci are most commonly found in the skeletal muscle, subcutaneous tissue, eye and brain [2]. The most common location in the eye is in the vitreous cavity or subretinal space [3]. While the identification of the characteristic clear cyst containing a white scolex is essentially diagnostic, intense vitreous inflammation may preclude direct visualization and B-scan ultrasound is often useful [4]. This report describes the management of two cysts in the posterior segment, an intravitreal and a subretinal cyst with evaginated scolex in the same eye.

## Case description

A 20-year-old vegetarian male with no ocular or systemic comorbidities presented to us with complaints of a painful diminution of vision in the right eye for the past month. He had been diagnosed with posterior uveitis and prescribed oral steroids elsewhere. On examination, the patient was noted to have reduced visual acuity in right eye (hand movement) compared with his left eye (20/20). The examination of the right eye revealed limbal congestion, 1^+^ cells in anterior chamber, and pigment dispersion over anterior lens capsule with clear lens. Fundus examination showed dense vitreal strands with no view of the retina. Ocular movements, lids and adnexa examination were unremarkable. Intraocular pressure was 16 and 18 mmHg in right eye (OD) and left eye (OS) respectively. 

## Investigations

**Figure 1:**
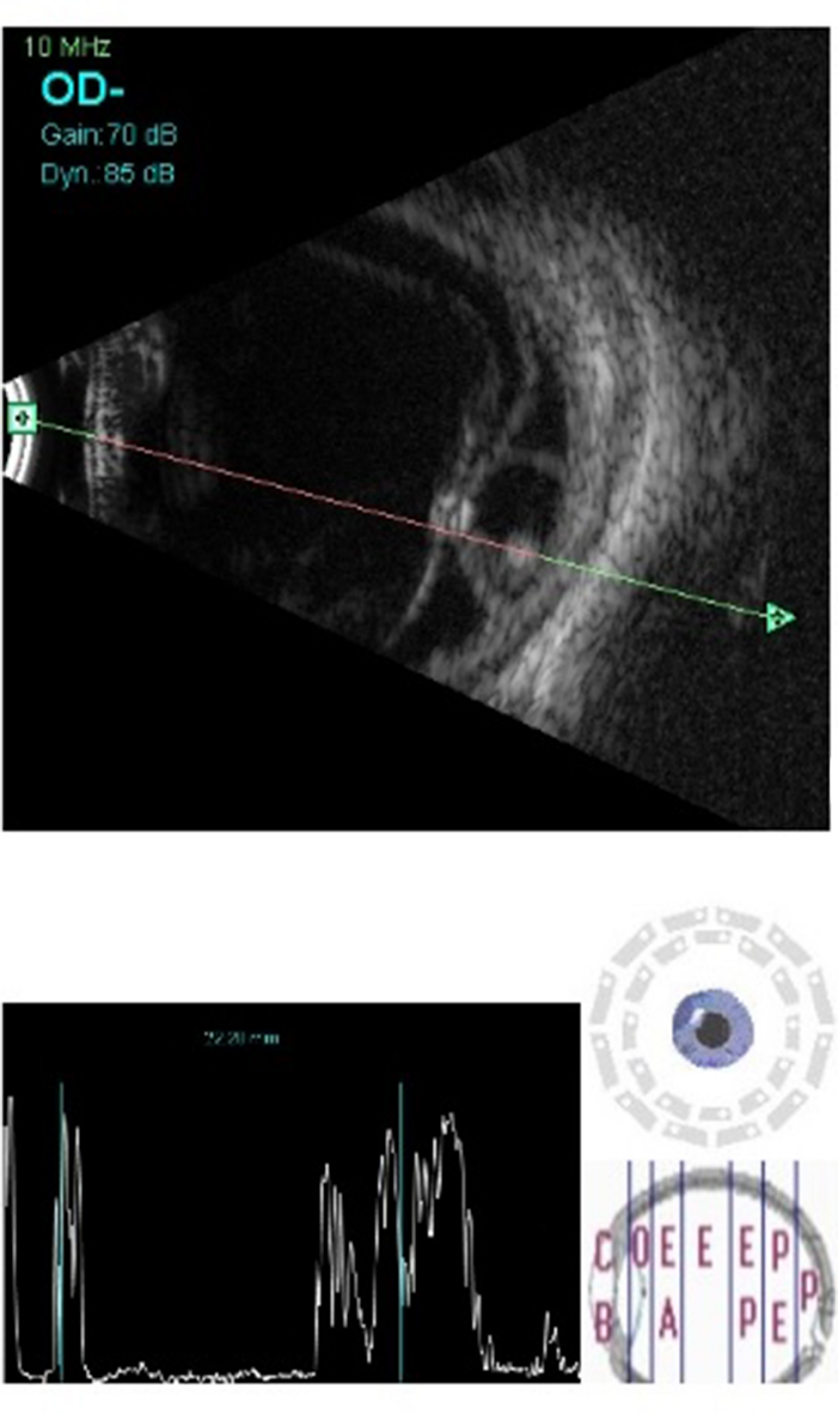
B-scan ultrasound of the posterior segment of the right eye showing inferotemporal retinal detachment with a well-defined subretinal cyst having high amplitude, dot-like echo suggestive of scolex. Hyperechoic dot with high amplitude spike in vitreous cavity suggestive of another scolex

**Figure 2:**
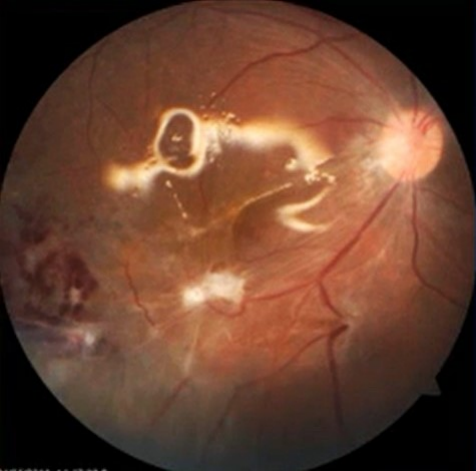
Two weeks post-operatively, showing attached retina, chorioretinal scar inferiotemporal to fovea, superficial haemorrhages corresponding to the area of exudative retinal detachment and subretinal cyst with silicon oil in situ

OD B-scan showed multiple membrane echoes of moderate to high intensity in the anterior vitreous with a hyperechoic central shadow moving with movement of the eye ball suggestive of dense vitreous membranes with scolex, an area of high intensity echoes at low gain at inferior temporal quadrant suggestive of retinal detachment and underneath it, a well-defined cystic lesion with clear contents and a hyperechoic shadow suggestive of a scolex (Figure 1 (Fig. 1) and Figure 2 (Fig. 2)). Orbital ultrasonography (USG) revealed no cystic lesions in the orbit or extraocular muscle. Stool examination, ELISA for serum antibodies (IgG) and CT scan brain was done, and neurocysticercosis was ruled out.

## Treatment

The patient underwent 23 gauge pars plana vitrectomy (PPV). We noticed two cysts with scolices, one oblong vitreal cyst enveloped by dense vitreous membranes in anterior vitreous and another subretinal spherical cyst of 5–6 disc diameter (DD) with overlying localised exudative retinal detachment in the infero-temporal quadrant. The macula was attached with retinal pigment epithelial changes. The vitreal cyst with scolex was cut and aspirated with a cutter. While inducing posterior vitreous detachment at this stage multiple retinal tears occurred in the inferior retinal periphery. Meanwhile larva from the subretinal cyst got evaginated and its increased undulating movements were noticed under the effect of endolaser light over the macular area. A retinotomy was made with endocautery at the inferotemporal edge of the subretinal cyst over the area of exudative retinal detachment. The subretinal cyst with evaginated and moving larva was brought into vitreous cavity, in-toto with active extrusion and then removed with a cutter. An epiretinal membrane was noticed inferotemporal to fovea with an adjacent area of chorioretinal scar. Using brilliant blue green (BBG) 0.5% dye, internal limiting membrane peeling was attempted and completed in a piecemeal fashion over the fovea. Fluid-air exchange, endolaser was done and silicon oil (1,000 centistroke) was injected. Postoperatively the patient was started on oral steroids (1 mg/kg body weight) and advised to maintain prone positioning for two weeks (Figure 2 (Fig. 2)). After two months of surgery, the patient was maintaining a best corrected visual acuity (BCVA) of 20/125 in OD with silicon oil in situ. The retina was attached with good laser marks and there was epiretinal membrane and a chorioretinal scar inferior temporal to fovea.

## Discussion

Our patient showed 1^+^ cells in the anterior chamber with dense vitreal strands. Wender et al. reported a positive correlation between the severity of inflammation and duration of indwelling cyst with or without intact cyst wall [5]. In a study reported by Kruger-Leite et al., 35% of the cysts were found in the subretinal space, 22% in the vitreous, 22% in the subconjunctival space, 5% in the anterior segment, and only 1% in the orbit [6]. The parasite is brought via the posterior ciliary arteries to the subretinal space usually in the region of the posterior pole due to rich blood supply. Thereafter it can enter the vitreous through a break in the overlying neurosensory retina causing rhegmatogenous retinal detachment, a macular hole or more commonly a healed scar [7]. We had a cyst with evaginated and freely moving larva in the subretinal space with an overlying retinal detachment and another cyst in the vitreous cavity with a small area of chorioretinal scarring giving rise to speculation of migration of one cyst from the subretinal space into the vitreous cavity.

In the era of microincision vitrectomy surgery (MIVS), a removal of the cysts using the vitreous cutter is advocated. The toxins released by the cyst frequently induce an inflammatory reaction with vitritis, uveitis, and sometimes endophthalmitis, particularly when the larva dies and liberates large amounts of toxins [6]. The high speed cutting rates with the maximum suction ensures that the cyst contents barely come in contact with the ocular structures with minimum release of toxins. Systemic corticosteroids are used before and after surgical removal of the cysticercosis cyst and help in reducing the worsening of inflammation induced by the death of the parasite [8].

The improvement of visual acuity in our case can be attributed to the removal of dense vitreal strands, the extramacular location of subretinal cyst, prompt and meticulous surgery and the use of oral corticosteroids in the immediate postoperative period. 

Key points of this report:

- Coexistence of twin cysts (intravitreal along with subretinal) in the same eye is possible with a chorioretinal scar and no visible break in retina.
- A high index of suspicion, prompt surgery employing MIVS and postoperative use of systemic corticosteroids paved the way for improvement of visual acuity in our case.

## Notes

### Acknowledgements

The authors would like to thank Mr. Lokesh Chauhan for his technical support.

### Competing interests

The authors declare that they have no competing interests.
